# Overexpression of rice gene *OsATG8b* confers tolerance to nitrogen starvation and increases yield and nitrogen use efficiency (NUE) in Arabidopsis

**DOI:** 10.1371/journal.pone.0223011

**Published:** 2019-09-25

**Authors:** Xiaoxi Zhen, Fan Xu, Wenzhong Zhang, Nan Li, Xin Li

**Affiliations:** 1 Rice Research Institute of Shenyang Agricultural University, Key Laboratory of Northern Japonica Rice Genetics and Breeding, Ministry of Education and Liaoning Province, Key Laboratory of Northeast Rice Biology and Genetics and Breeding, Ministry of Agriculture, Shenyang, China; 2 Shen Yang Product Quality Supervision and Inspection Institute, Shenyang, China; National Taiwan University, TAIWAN

## Abstract

Nitrogen (N) is an important element required for plant growth and development, which also affects plant yield and quality. Autophagy, a conserved pathway in eukaryotes, degrades and recycles cellular components, thus playing an important role in N remobilization. However, only a few autophagy genes related to N remobilization in rice (*Oryza sativa*) have been reported. Here, we identified a core autophagy gene in rice, *OsATG8b*, with increased expression levels under N starvation conditions. It was investigated the function of *OsATG8b* by generating three independent homozygous *35S-OsATG8b* transgenic *Arabidopsis thaliana* lines. The overexpression of *OsATG8b* significantly enhanced autophagic flux in the transgenic Arabidopsis plants. It was also showed that over-expressing *OsATG8b* promoted growth and development of Arabidopsis, in which the rosette leaves were larger than those of the wild type (WT), and the yield increased significantly by 25.25%. In addition, the transgenic lines accumulated more N in seeds than in the rosette leaves. Further examination revealed that overexpression of *OsATG8b* could effectively alleviate the growth inhibition of transgenic Arabidopsis under nitrogen (N) stress. N partitioning studies revealed that nitrogen-harvest index (NHI) and nitrogen use efficiency (NUE) were significantly increased in the transgenic Arabidopsis, as well as the ^15^N-tracer experiments revealing that the remobilization of N to seeds in the *OsATG8b*-overexpressing transgenic Arabidopsis was high and more than WT. Based on our findings, we consider *OsATG8b* to be a great candidate gene to increase NUE and yield, especially under suboptimal field conditions.

## Introduction

Nitrogen (N) is one of the most important nutrient elements for plants and is required for growth and development. N is also a vital component of amino acids, protein, nucleic acid, chlorophyll, and plant hormones [1]. In plants, especially cereal crops, the yield and quality of the grain depends not only on the absorbed N prior to flowering, but also on the mobilization of reused N from the mature leaves during senescence [2,3]. Plants are sedentary and cannot move to acquire nutrients and minerals as needed. Survival depends on their ability to consume the mineral nutrients available in the rhizosphere, metabolize, recycle, and conserve them efficiently during their lifespan [4,5]. The chloroplasts are the main source of C and N recycling in plants, which contribute to the 75–80% nitrogen of the leaf total protein in C3 plants [6]. Rubisco, a photosynthetic CO_2_ fixing enzyme, accounts for more than 50% of the soluble protein content in the leaves, and is the main source of nitrogen mobilization and reuse [7]. In the later stages of development, leaf protein (especially the chloroplast protein) is rapidly degraded as the output of nitrogen, as the released free amino acids and other compounds are transferred to the developing reproductive and storage organs, such as new leaves or seeds [2,8]. This mobilized nitrogen is an important source of nitrogen for seeds [9]. The circulation process for organic nitrogen transfer from aging tissues to seeds in plants is an important determinant of productivity and yield, especially under nitrogen deficiency stress [7]. Although the pathways and steps in protein turnover in aging leaves are not clear, plastid resident proteases, senescence-associated vacuoles (SAVs), and macroautophagy (hereafter referred to as autophagy) are considered to be three important routes [4,10].

Autophagy, which is a conserved vacuolar degradation pathway by which cells recycle components, including unwanted macromolecular substances or damaged organelles, plays a vital role in nutrient remobilization. Autophagy is continuously maintained at a basal level to maintain cell homeostasis under normal conditions. In response to stress or nutrient starvation, autophagy is enhanced to facilitate the degradation of increasing levels of toxic and damaged components, and nutrients mobilized from this recycled cell material is then used for the maintenance of cellular processes and adaptation to stress [11,12]. A pioneering study by Professor Yoshinori Ohsumi led to the discovery of the AuTophaGy-related genes (*ATG* genes) in yeast [13]. A total of 16 *ATG* genes were identified in the first yeast screening. Subsequently, many *ATG* genes have been characterized in different species, including mammals and plants [14,15]. In Arabidopsis (*Arabidopsis thaliana*), most *ATG* genes are transcriptionally up-regulated during leaf senescence and nutrient starvation [16,17]. Early senescence symptoms and hypersensitivity to C and N starvation were displayed in autophagy mutants [18], and a previous study revealed that the overaccumulation of salicylic acid (SA) is involved in the early senescence in *atg* mutants [19]. A study of Arabidopsis *atg* mutants provide the first evidence that N remobilization from the leaves to the seeds is controlled by autophagy [20]. In Arabidopsis, the wild type (WT) and *atg* mutants (*atg18a* RNAi, *atg5* and *atg9*) were fed with ^15^NO_3_^-^ during the vegetative growth stage, ^15^N remobilization sharply decreased in all the *atg* mutants compared to the WT under N-limited conditions, and the NUE decreased significantly to about 50% in the seeds. In the rice *Osatg7-1* mutant, biomass and NUE during the vegetative growth stage were significantly reduced, and the mutant was unable to mobilize N in aging leaves [21]. In maize (*Zea mays*), ^15^N pulse-chase analysis revealed that the amount of recycled N was reduced by two-fold in the *atg12* mutant compared with that of the WT [22].

Among the many plant ATG proteins, the abundance of ATG8 proteins at the cellular level can regulate the size of autophagosome. ATG8 is a ubiquitin-like protein conjugated to phosphatidylethanolamine (PE) on the autophagic membrane. ATG8 plays a central role in autophagy, which is involved in autophagic vesicle expansion [23,24] and is located on the autophagosome membrane [25]. The ATG8 is often used as a reliable marker of autophagic activity in plants and animals [12,26]. While ATG8 protein is unique in yeast, many isoforms exist in plants and are encoded by different genes. There are 7 identified ATG8 isoforms (*OsATGa* to *OsATGg*) in rice genome. *OsATG8a* was the first cloned rice *ATG8* gene, interacted with *OsATG4* [27]. *OsATG8a*, *OsATG8b* and *OsATG8c* share high levels of amino acid sequence similarity, while *OsATG8d* has high similarity to *AtATG8i* [28,29]. In addition, *OsATG8a* and *OsATG8c* had more closely phylogenetic relationships with *ZmATG8a* and *ZmATG8c*, respectively [30]. However, *OsATG8e* lacks supporting expressed sequence tag (EST) data, *OsATG8f* has no corresponding full-length cDNA, and *OsATG8g* has not been mapped to the rice genome [29]. Several studies indicate that overexpression of *ATG8* promotes plant growth and increases tolerance to N starvation in plants. For example, transgenic Arabidopsis plants overexpressing *AtAtg8f* were slightly larger in size than the control plants, and flowered earlier than the control plants in a N-deficient medium [31]. Constitutive expression of apple *MdATG8i* and soybean *GmATG8c* in apple and soybean calli, respectively, not only led to better tolerance to N starvation, but also accelerated the growth of calli under optimal growth conditions. Overexpression of soybean and apple *ATG8* genes, *GmATG8c* and *MdATG8i*, respectively, promoted growth and improved the yield in the transgenic Arabidopsis [30,32]. Similarly, Li et al observed that heterologous expression of foxtail millet *SiATG8a* in Arabidopsis and rice improved their performance under normal growth conditions and conferred tolerance to N starvation [33,34]. Recently, research of Masclaux-Daubresse’s group showed that overexpression of *AtATG8*s (*AtATG8a*, *AtATG8e*, *AtATG8f*, *AtATG8g*, respectively) in Arabidopsis increased autophagosome number and stimulated autophagic activity, increased N remobilization efficiency (NRE) only under full N conditions but did not affect yield and biomass [35].

In order to screen for potential yield and N remobilization genes in rice, the rice AT*G8* gene *OsATG8b* (LOC_*Os04g53240*) was identified. *OsATG8b* has a 360-bp coding sequence (CDS) and encodes 120 amino acids. We then confirmed its function using *35S-OsATG8b* in transgenic Arabidopsis plants. Compared to the WT, three independent transgenic lines were found to have improved vegetative growth, developed to the reproductive stage slightly earlier, were taller, and produced more siliques and seeds. Overexpression of *OsATG8b* therefore, not only led to better performance under normal growth conditions but also maintained growth under N starvation. The autophagic flux was increased in the transgenic lines. At the same time, the number of autophagosomes were significantly enhanced under N deficiency (N free with 0 mM nitrogen) with or without the addition of the autophagy inhibitor. Growth inhibition caused by nitrogen stress was effectively alleviated. N partitioning studies have shown that overexpression of *OsATG8b* increased N allocation to seeds and have enhanced NHI and NUE indicated by NHI/HI ratio [20], while the ^15^N allocation to seeds was significantly higher than that in the WT using ^15^N isotope tracer experiment. Therefore, our results indicate that *OsATG8b* may be an important candidate gene for rice with synergistically-enhanced NUE and better yield potential.

## Results

### *OsATG8b* displays N stress inducible expression in rice and the expression patterns of *OsATG8b* in transgenic Arabidopsis

**Fig 1:**
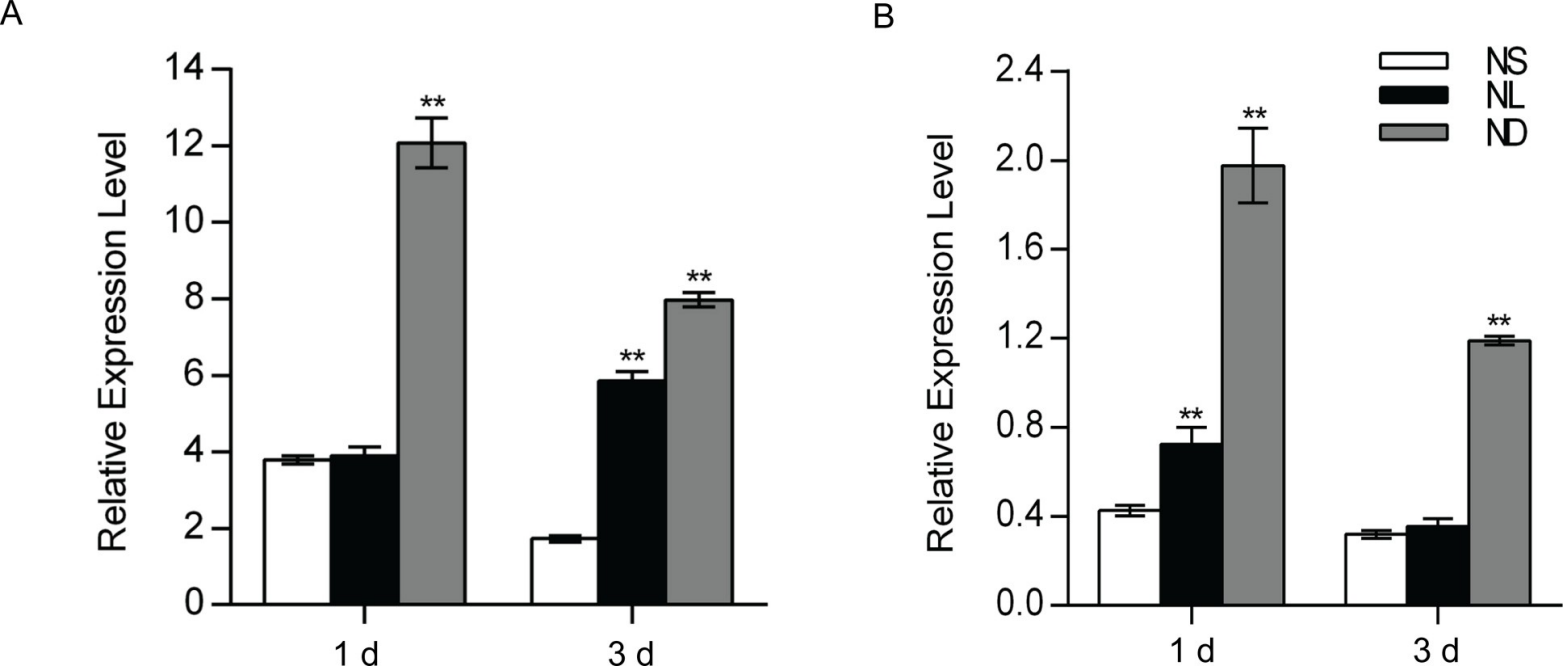
Identification of *OsATG8b* as a N deficiency inducible gene of rice by real-time RT-PCR. The rice seeds were sterilized and germinated at 28°C for 2 d, then seedlings cultured with half Hoagland’s N-sufficient solution for 14 days, and transferred to the same N-sufficient (NS, 3.5 mM N) solution as the control, low N (NL, 0.8 mM N) solution and the N-free (ND, 0 mM N) solution for 1 d and 3 d. (A) The expression of *OsATG8b* gene in leaves. (B) The expression of *OsATG8b* gene in roots. *OsActin1* was used as an internal control. Values are means ± SD of three biological replicates, *P < 0.05, **P < 0.01 (t-test).

In order to analyze whether the expression of *OsATG8b* in rice is responsive to N limitation, we first examined the transcript levels of *OsATG8b* by real-time RT-PCR. When rice seedlings were placed under N stress for 1 day, the expression level of *OsATG8b* in leaves was not different in low N (NL) conditions when compared with that of the control, but was highly induced under N deficient (ND) conditions (Fig 1A). In addition, the levels of *OsATG8b* in the root increased with increasing degree of N stress (Fig 1B). After 3 days of N stress, the expression of *OsATG8b* in leaves increased with increasing degree of N stress but was highly induced only under ND conditions in the root (Fig 1). These results suggest that expression of *OsATG8b* in rice is induced by N stress.

To examine the temporal and spatial expression patterns of *OsATG8b*, we performed histochemical analysis of the promoter-GUS fusion report systems. We transformed Arabidopsis with the *Pro*_*OsATG8b*_*-GUS* construct, and histochemical staining indicated that GUS activity could be detected in the germinating seed, cotyledon, hypocotyl, the rosette leaf, root, as well as the mature inflorescence and silique in the transgenic seedlings (S1 Fig).

### Overexpression of *OsATG8b* in transgenic Arabidopsis promotes growth and increases yield

**Fig 2:**
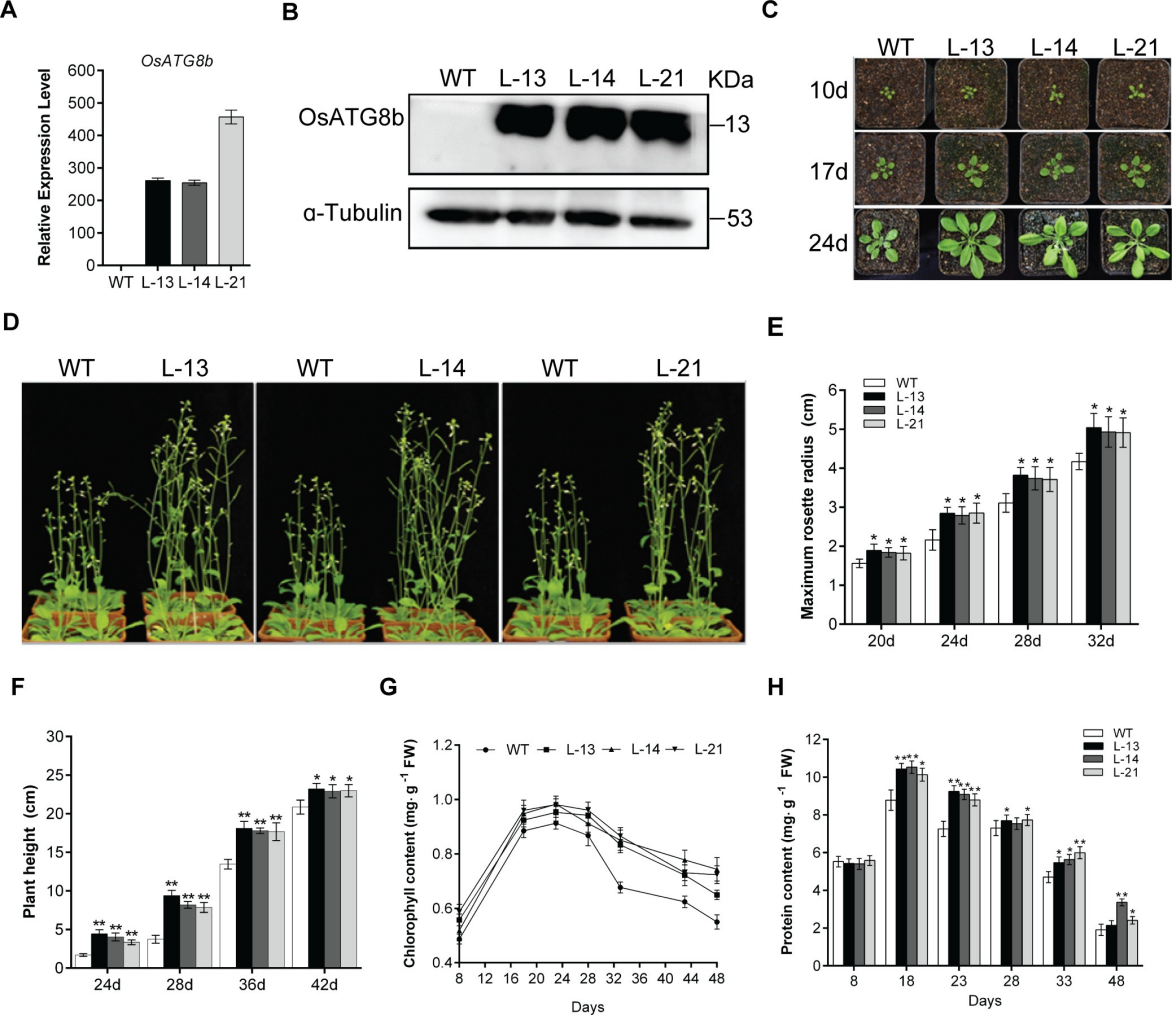
Overexpression of *OsATG8b* promotes growth of transgenic Arabidopsis. 8-day-old seedlings were transferred to soil and cultivated under a long-day photoperiod. (A) Expression level of *OsATG8b* in 14-day-old seedlings of transgenic lines and WT. (B) Immunoblot analysis of the accumulation of OsATG8b in 14-day-old seedlings of the WT and *35S-OsATG8b* transgenic lines (L-13, L-14, L-21) with a polyclonal anti-OsATG8b antibody. Equal protein loads were confirmed by immunoblot analysis with an α-tubulin antibody. (C) Panels from top to bottom show phenotypic observations of transgenic lines and WT at 10 days, 17 days and 24 days after transfer to soil, respectively. (D) Side view of transgenic lines and WT at 42 days after transfer to soil. (E)-(H) The maximum rosette radius, plant height, chlorophyll content and soluble protein content of transgenic lines and WT. Values are means ± SD of three biological replicates (n = 24), *P < 0.05, **P < 0.01 (t-test).

**Fig 3:**
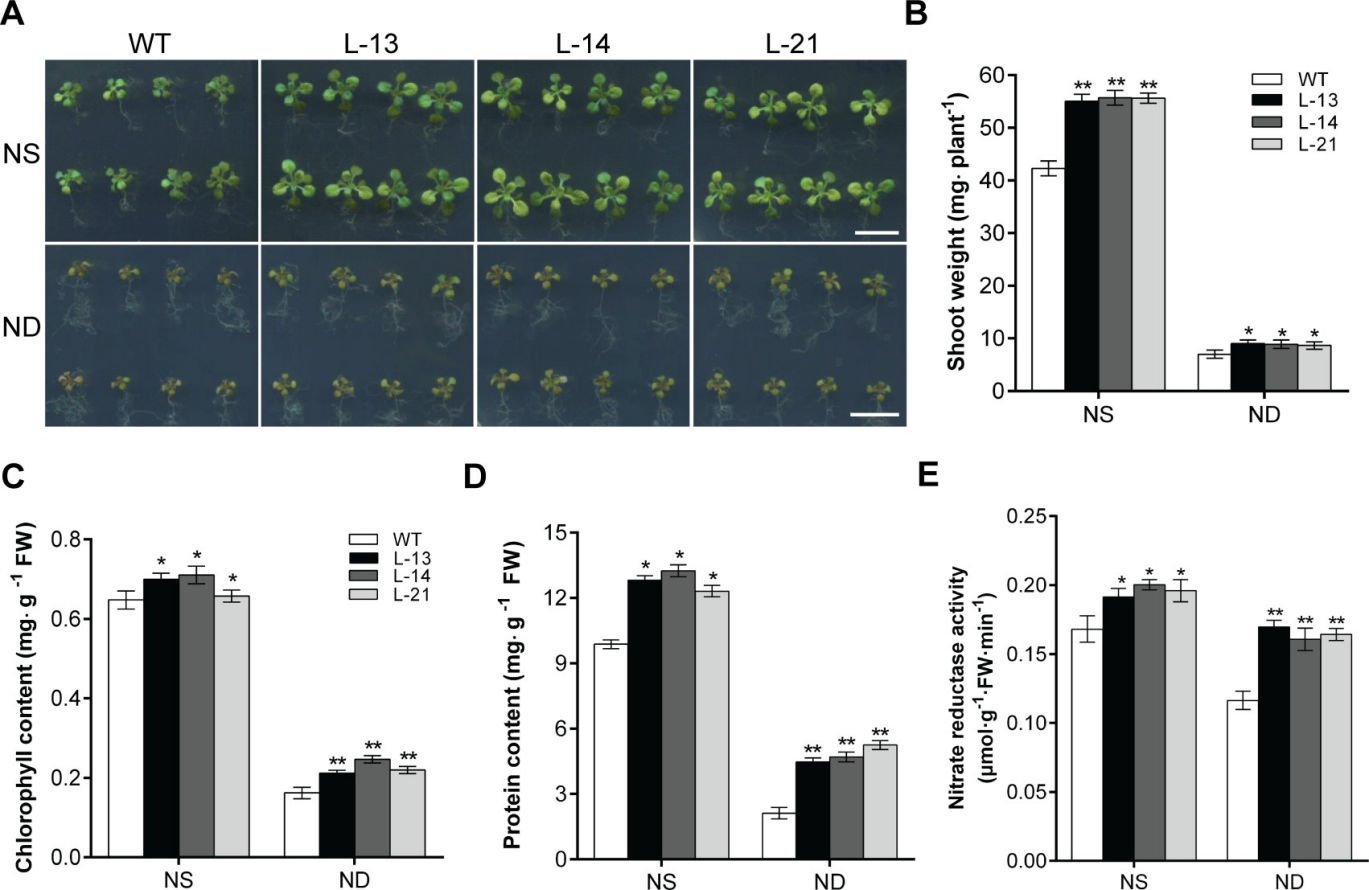
Overexpression of *OsATG8b* enhances tolerance to N deficiency in transgenic Arabidopsis. (A) 7-day-old seedlings of transgenic lines and WT were transferred to ½ MS medium with sufficient (NS) or deficient (ND) N for 9 days. (B) The shoot weight of WT and transgenic Arabidopsis lines under NS or ND. (C) and (D) The chlorophyll content and soluble protein content in rosette leaves of transgenic lines and WT under NS or ND. (E) The nitrate reductase (NR) activity in rosette leaves of transgenic lines and WT under NS or ND. Bar = 1 cm. Values are means ± SD of three biological replicates (n = 16), *P < 0.05, **P < 0.01 (t-test).

To fully understand the functional roles of *OsATG8b*, we transformed Arabidopsis plants with the *35S-OsATG8b* binary expression vector. We obtained over 10 independent transgenic lines. In order to eliminate the influence of *AtATG*s, real-time RT-PCR was used to monitor the transcript levels of *AtATG*s. We found that the transcript levels of the endogenous *AtATG*s were unchanged and not affected in the transgenic lines (S2 Fig). The transcript and protein levels of *OsATG8b* were confirmed in three homozygous transgenic lines using PCR, real-time RT-PCR and western blotting (Figs 2A, 2B and 3). Lines 13, 14 and 21 (L-13, L-14, L-21) refer to the over-expressed lines. Phenotypic effects of *OsATG8b* were observed throughout the plant developmental stages. Under normal growth conditions, the rosette of the transgenic lines was significantly larger and grew rapidly than the WT (Fig 2C and 2E). Overexpression of *OsATG8b* accelerated plant development, bolting as flowering occurred 6 days earlier and also produced more total leaves in the transgenic lines when compared to that in the WT (Table 1), while the leaf number at bolting was not significant different with WT. On an average, plant height in the transgenic lines was significantly increased by up to 10.42% when compared to the WT plant height (Fig 2D and 2F). The chlorophyll content of the rosette leaves in the transgenic lines was consistently higher than those seen in the WT as the seedlings aged (Fig 2G). There was minimal or almost no difference in the soluble protein content in the rosettes of 8-day-old transgenic seedlings and the WT seedlings; however, from 8 days until plant maturity, the soluble protein content in the transgenic lines was consistently higher than those seen in the WT plants (Fig 2H). We further investigated the effect of *OsATG8b* on yield and found that the transgenic lines produced significantly higher number of siliques. As a result, the transgenic lines had a significantly higher thousand grain weight and total seed weight per plant than those in the WT (Table 2). These results show that constitutive expression of *OsATG8b* both promotes growth and increases yield in transgenic Arabidopsis.

**Table 1:** Total leaf number, Bolting and flowering times of WT and *35S-OsATG8b* transgenic Arabidopsis.

	WT	L-13	L-14	L-21
Total leaf number	14.12 ± 0.35	15.06 ± 0.47**	15.19 ± 0.52**	15.28 ± 0.58**
Bolting time (d)	36.56 ± 1.58	30.78 ± 2.07**	31.39 ± 1.91**	31.06 ± 2.01**
Flowering time (d)	42.67 ± 1.75	35.94 ± 1.98**	36.61 ± 1.94**	36.44 ± 2.01**

Values are means ± SD of three biological replicates (n = 24)

**P < 0.01 (t-test), significant difference from the WT, the time to flowering and bolting were scored as days after sowing (DAS). The total leaf number was counted after the rosettes growth completed.

**Table 2:** Yield-related characteristics of WT and *35S-OsATG8b* transgenic Arabidopsis.

	WT	L-13	L-14	L-21
Total number of siliques	95.74 ± 3.86	139.26 ± 3.13**	147.22 ± 3.62**	140.74 ± 3.48**
Yield per plant (mg)	132.34 ± 7.89	160.13 ± 6.02**	172.77 ± 5.76**	164.92 ± 5.13**
Thousand grain weight (mg)	14.87 ± 0.23	16.36 ± 0.21**	17.54 ± 0.41**	16.31 ± 0.24**

All seeds on a single-plant was harvested individually and the yield per plant was expressed as the weight of total seeds per plant, values are means ± SD of three biological replicates (n = 10)

**P < 0.01 (t-test).

### Overexpression of *OsATG8b* in transgenic Arabidopsis enhances tolerance to N deficiency

To investigate the effect of *OsATG8b* overexpression on the plant responses to N stress, seven-day old transgenic seedlings and WT were transferred to ½ MS medium with sufficient or free N and incubated for 9 days. Changes to leaves and root were closely monitored. The transgenic lines overexpressing *OsATG8b* performed better than the WT, both under N-sufficient and N-free conditions. The transgenic lines had significantly larger rosettes (Fig 3A), as also the fresh weight of leaves in the transgenic lines was significantly higher both under N-sufficient and N-deficient conditions when compared to the WT (Fig 3B). The chlorophyll and soluble protein contents were also significantly higher than that of the WT (Fig 3C and 3D), and the enzymatic activity of nitrate reductase (NR) in transgenic Arabidopsis was obviously increased (Fig 3E), whether the supply of N was sufficient or deficient. These results indicate that the transgenic lines were able to tolerate N deficiency better and improve the N assimilation efficiency.

### Overexpression of *OsATG8b* in transgenic Arabidopsis enhances the autophagic activity and flux

**Fig 4:**
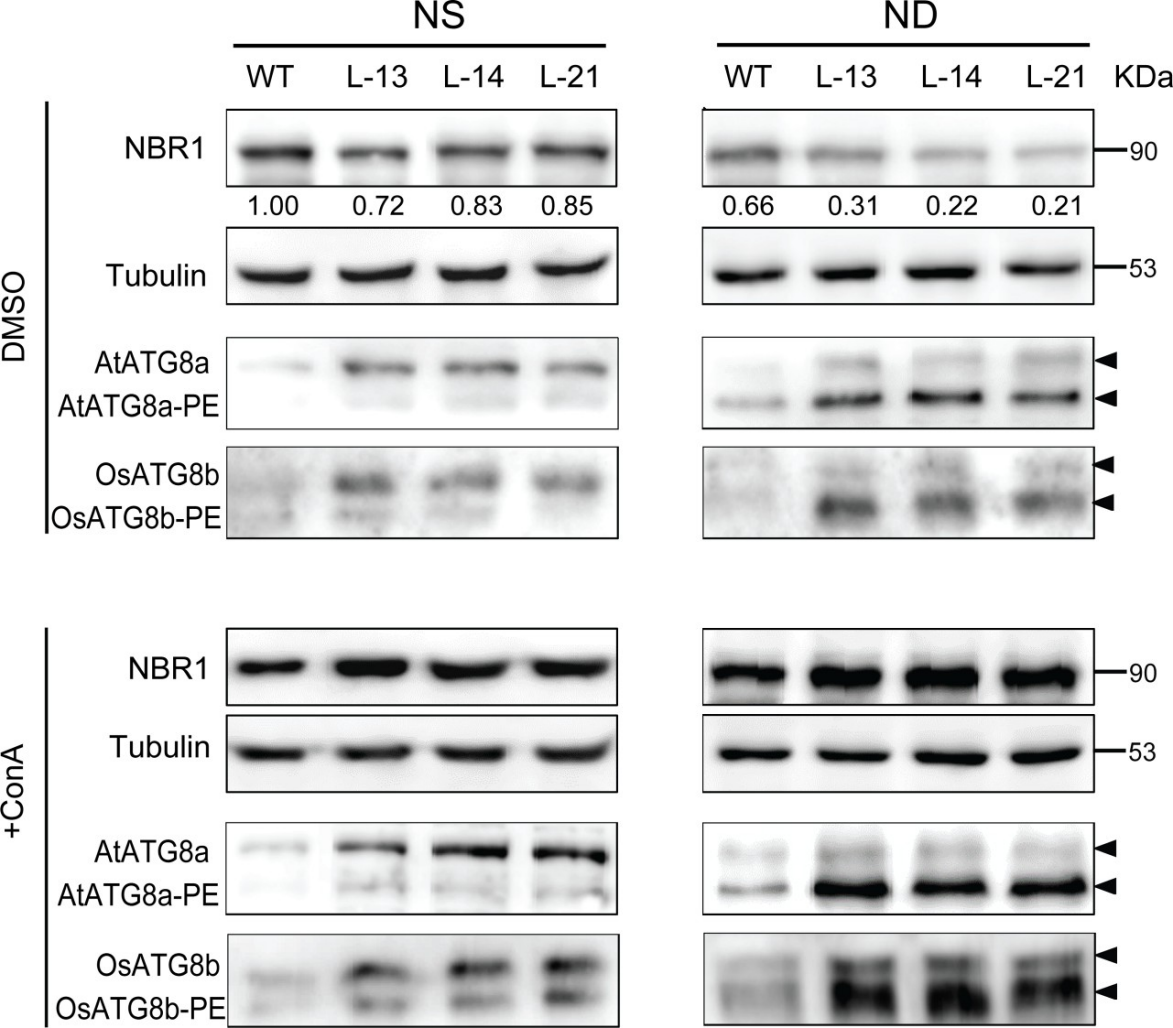
Overexpression of *OsATG8b* enhanced the autophagic flux in transgenic Arabidopsis. 9-day-old seedlings of transgenic lines and WT were transferred to NS or ND medium with 0.5 μM ConA or solvent control DMSO for 24h. Immunoblot analysis the accumulation of NBR1 with anti-Arabidopsis NBR1, near equal protein loads were confirmed by immunoblot analysis with an α-tubulin antibody. The membrane fraction was used to detect the level of lipidated (ATG8-PE) and free ATG8 with Arabidopsis anti-AtATG8a or an anti-OsATG8b antibody. The number under the blot indicated the intensity ratios of the NBR1 protein in each line after analyzed by Image J and normalized to the WT under NS condition (value set to 1).

To investigate whether the autophagy activity is enhanced in the transgenic Arabidopsis overexpressing *OsATG8b*, seven-day-old seedlings of transgenic lines and WT were treated with ND for 12 h, and MDC staining was used to determine autophagic activity in the leaves. Concanamycin A (ConA) enables distinct observation of the autophagosome by increasing the internal pH in the vacuolar and resident hydrolase activity in an optimal environment [36,37]. The autophagy receptor NBR1 (Neighbor of BRCA1) protein of Arabidopsis is an autophagy substrate that degrades in the vacuole, and the degradation of NBR1 can be used as a measure of selective autophagic flux in plants [38,39]. As shown in Fig 4, the consumption of NBR1 protein was higher in transgenic lines, under NS condition, the expression level of NBR1 protein in transgenic lines were 15%-28% less than that of WT. While the degradation of NBR1 protein was 34% in WT under ND compared to NS condition, but it was as much as 41%-64% in transgenic lines (Fig 4, DMSO-ND). However, when applied with ConA to block the degradation of autophagic bodies and NBR1 protein, the accumulation of NBR1 protein was greatly increased (Fig 4, +ConA), especially under the ND treatment. The previous reports showed that the lipidation of ATG8 can be used to indicate the autophagic activity [40,41]. As the autophagy core protein ATG8a usually acts as the autophagic flux marker protein, and previous studies have used Arabidopsis anti-ATG8a antibody to exam the autophagic flux in various species (such as Arabidopsis, maize and tomato) [22,40–42]. The autophagy flux in our transgenic plants was monitored with both anti-AtATG8a and anti-OsATG8b antibodies. These results showed that overexpression of *OsATG8b* significantly increased the accumulation of both AtATG8a-PE and OsATG8b-PE in transgenic lines, thus the lipidation of OsATG8b was enhanced, especially under N deficiency condition (Fig 4, DMSO), and increased to a greater extent after ConA treatment (Fig 4, +ConA). The detection signal of lipidation used by anti-OsATG8b antibody was better than that of anti-AtATG8a antibody.

**Fig 5:**
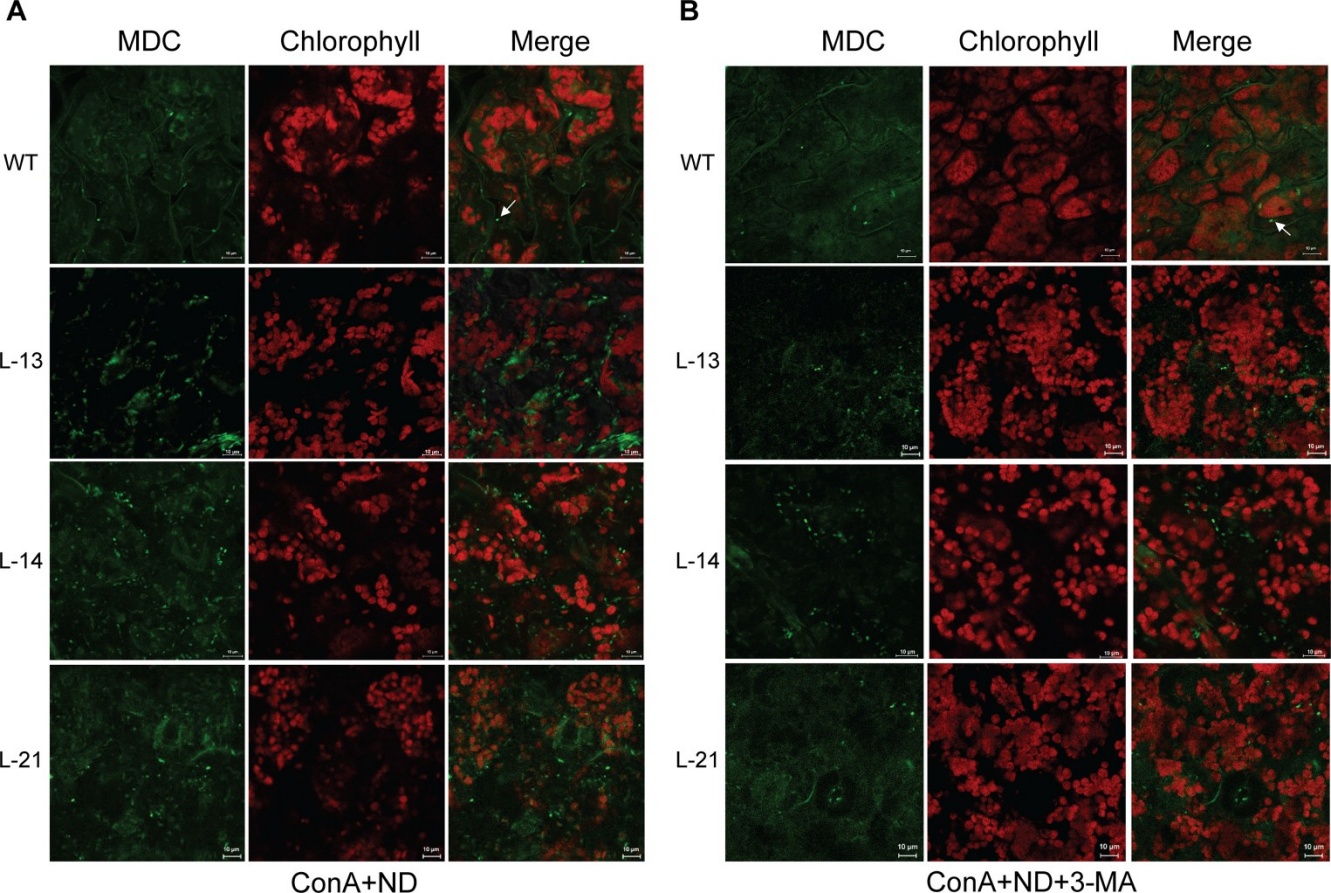
Overexpression of *OsATG8b* in Arabidopsis enhances the autophagic activity under N deficient condition. (A) 7-day-old seedlings of transgenic lines and WT were transferred to in ND liquid medium with 1 μmol·L^-1^ ConA for 12h, MDC-stained autophagosomes in leaves were observed by confocal microscopy. (B) 7-day-old seedlings of transgenic lines and WT were transferred to ND liquid medium containing 1 μmol·L^-1^ ConA with 5 mM 3-MA for 12h, MDC-stained autophagosomes in leaves were observed by confocal microscopy.

Monodansylcadaverine (MDC), an acidophilic dye widely used in mammals and plants can be used as a probe to detect autophagosome [42]. To facilitate the observation of autophagic bodies, the H^+^-ATPase inhibitor ConA was added. The leaves of transgenic Arabidopsis and WT were excised and immediately observed by confocal microscopy. We observed significantly more MDC-positive autophagic structures in the mesophyll cells of the transgenic lines, and the fluorescence signal intensity was higher than that seen in the WT (Fig 5A). The autophagy inhibitor 3-methyladenine (3-MA), specifically inhibits PI3K activity [43,44]. The addition of 5 mM of 3-MA to the N-deficient treatment clearly suppressed the fluorescence signal of MDC-stained autophagosomes, but some fluorescent signal could still be observed in the transgenic lines (Fig 5B). The fluorescence ratio (positive signal/aera) in the WT was 30.87 under ND condition, and decreased to 13.07 after 3-MA treated, while the ratios in transgenic lines were 121.85 and 62.53. It was showed that the fluorescence ratios in the overexpressing lines were up to 3 or 4 times more than that in WT with or without 3-MA. All these results reveal that overexpression of *OsATG8b* could significantly increase the autophagosome number and autophagic flux in transgenic Arabidopsis. This can effectively improve the tolerance of transgenic Arabidopsis to N deficiency stress.

### Overexpression of *OsATG8b* in transgenic Arabidopsis modifies N use efficiency and N remobilization efficiency

**Fig 6:**
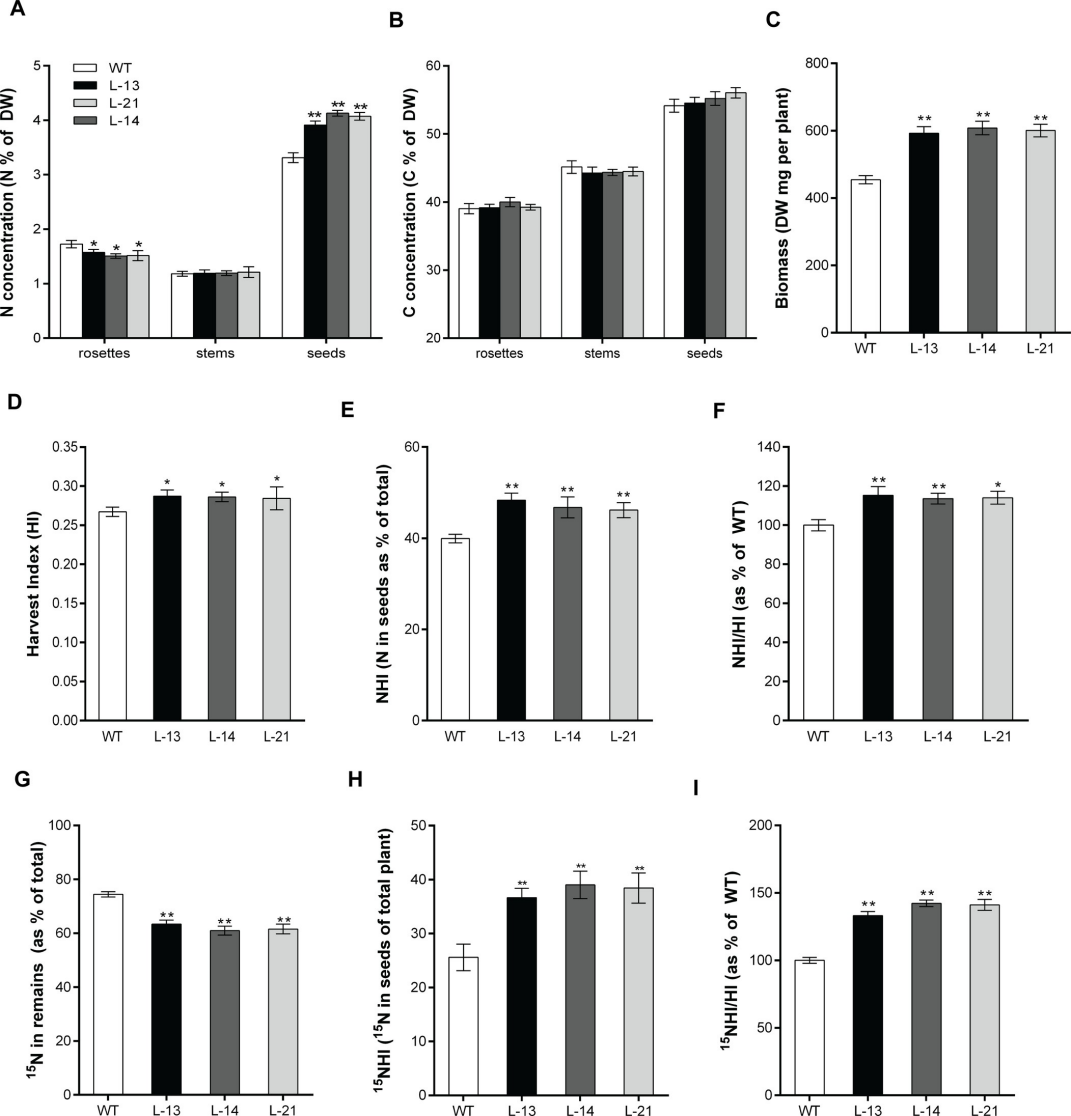
Evaluation of NUE and NRE in transgenic Arabidopsis. (A) N concentration (N%, presented as mg per 100 mg DW) and (B) C concentration (C%, presented as mg per 100 mg DW) of the transgenic lines and WT were measured in rosettes, stems and seeds after harvesting. (C) Biomass accumulation as measured by dry weight of the whole plant. (D) HI was measured as the ratio of the dry weight of total seeds to the dry weight of remains. (E) NHI was measured as the partitioning total N in seeds. (F) NHI/HI ration was calculated as a percentage of WT and used to estimate NUE between the transgenic lines and WT. (G) Partitioning of ^15^N in remains of the transgenic lines and WT after harvesting. (H) ^15^NHI was measured as the partitioning total ^15^N in seeds. (I) ^15^NHI/HI ration was calculated as a percentage of WT and used to estimate NRE between the transgenic lines and WT. Values are means ± SD of three biological replicates (n = 12), *P < 0.05, **P < 0.01 (t-test).

To determine whether the overexpression of *OsATG8b* modifies the N status at the whole plant level, the N concentration in rosettes, stem, and seeds was measured after harvesting. The N concentration (N%) in rosettes of the transgenic lines was much lower than that of the WT, but there was no difference in the stem samples. In contrast, the N% in seeds was significantly higher in the transgenic lines (Fig 6A). However, there was no significant difference in the C concentration (C%) between the transgenic lines and the WT (Fig 6B). Given the increased yield in transgenic lines, we further analyzed the biomass of plants. Relative to WT, the biomass of the transgenic lines increased by 25.95% to 26.89% (Fig 6C).

We studied the effect of *OsATG8b* on plant yield, seed N filling, harvest index (HI) and N harvest index (NHI). While NHI is an important indicator of production, it also highlights the N allocation efficiency to seeds. As shown in Fig 6D and 6E, the HI and NHI were significantly higher in the transgenic lines compared to that in the WT plants. In order to determine the NUE, we used the NHI/HI ratio as an indicator of NUE variation [20]. The NHI/HI ratio in the transgenic lines was significantly higher than that of the WT (Fig 6F), suggesting that *OsATG8b* might be essential for both seed yield and NUE. These results clarify that overexpression of *OsATG8b* effectively redeploys N allocation from the leaves to seeds and increases the NUE of Arabidopsis plants.

To monitor N movements accurately, ^15^N-pulse chase experiments were performed at the bolting stage similarly as described in previous studies [20,22]. Samples of *OsATG8b*-overexpressing lines and WT were dissected into seed and remains (rosettes and stems), then traits for N utilization and remobilization were calculated. To determine the N remobilization efficiency (NRE), the partitioning of ^15^N in WT and transgenic lines were tracked. It was showed that overexpression of *OsATG8b* obviously reduced the partitioning of ^15^N into remains (Fig 6G). In contrast, the *OsATG8b*-overexpressing lines accumulated significantly more ^15^N in seeds relative to WT, as indicated by the higher ^15^NHI in transgenic lines (Fig 6H). The ratio of ^15^NHI to HI was next used to compare the NRE variation as described previously [20,22], and the ^15^NHI/HI were substantially increased in *OsATG8b*-overexpressing lines than in WT (Fig 6I), suggesting that through overexpression of *OsATG8b* could significantly improve the N remobilization into seeds, which resulted in favorably increased yield and NUE.

## Discussion

Autophagy is involved in removal of damaged proteins and organelles, and response to biological and abiotic stresses [11]. Previous studies have shown that nutrition-limitation induces autophagy in plants [45,46]. Under C and N stress conditions, transcription of many plant *ATG* genes was significantly up-regulated similar to the high autophagy activity [28,29,33,47,48]. The transcriptional level of *OsATG8b* increased with N deficiency in rice seedlings (Fig 1), which is evidence that *OsATG8b* response to N stress. On the other hand, it would provide evidence that overexpression of *OsATG8b* could effectively increase tolerance to N stress (Fig 3A). The GUS activity of *OsATG8b* promoter was detected in the inflorescence, seedpod, and in particular was higher in the aging leaves, which indicated that *OsATG8b* gene may participate in seed formation in plants and also be involved in autophagic degradation during leaf senescence (S1A and S1E Fig).

With defective autophagy, the redundant N in the cells cannot be effectively recovered or utilized, leading to nutrient accumulation, wastage, cell death, and lower amino acid content, the *atg* deletion mutants showed accelerated senescence, decreased survival rate and other unfavorable phenotypes under nutrient stress conditions [11,20–22,49,50]. Consistent with this view, the transgenic Arabidopsis plants containing *35S-OsATG8b* not only performed stronger and better than the WT under normal growth conditions, but also effectively overcome growth inhibition under N deficiency conditions (Fig 3A). In addition, we also found that the transgenic lines had increased chlorophyll content and soluble protein content in the leaves (Fig 2), so did the NR activity (Fig 3D). These results indicate that overexpression of *OsATG8b* leads to increased accumulation of available N in the ‘source’ of transgenic Arabidopsis. The overexpression of *OsATG8b* accelerated the switch from vegetative to reproductive growth in the transgenic Arabidopsis (Table 1), which was probably because of a healthy vegetative phase to have an extended reproductive phase resulting in increased number of siliques, and consequently, higher thousand grain weight and better yield (Table 2).

In the *OsATG8b*-overexpressing lines, the enhanced degradation of NBR1 and lipidation of ATG8, as well as the ConA-mediated increased accumulation of NBR1 and AtATG8a-PE/OsATG8b-PE, all these results revealing that the autophagic flux and activity significantly increased under ND condition (Fig 4-ND). Additionally, the MDC staining also supported this conclusion (Fig 5). Even under N sufficient condition, the nutrients still could be mobilized and reused from leaves to reproductive organs (seeds) to the maximum extent, as a result of enhanced autophagy activity (Fig 4-NS). The growth, development, and yield of transgenic Arabidopsis could be enhanced.

Autophagy is also as an important role in the remobilization and transfer of N to the grain [11,24]. Both NHI and seeds N concentration (N%_Seeds_) are major indicators of NUE and seed nutritional quality [7]. Overexpression of *OsATG8b* in transgenic Arabidopsis resulted in higher NHI, HI, NHI/HI, N%_Seeds_, and at the same time reduced N waste in their dry remains (Fig 6), indicating that *OsATG8b* plays a vital role in NUE. This could explain *OsATG8b* increases both grain production and the allocation of biomass to grain. On the other hand, ^15^NHI/HI ratio is used to measure the variations as Nitrogen Remobilization Efficiency (NRE). A significantly higher ^15^NHI/HI ratio observed in the transgenic lines compared to the WT (Fig 6I) implied that *OsATG8b* could also increase the allocation of N to seeds. The increased seed yield was at least partially caused by effective remobilization of assimilated N from the vegetative tissue to the developing seeds. Corresponding to the increased autophagy activity described above, since autophagy likely contributes to the nitrogen pool for seed production, we speculated that the transgenic Arabidopsis could increase N transported and accumulated during the vegetative growth stage, which in-turn could provide abundant nutrients for reproductive growth and higher yield even under N full condition.

A recent paper of Masclaux-Daubresse’s group has just reported that overexpression of four *AtATG8* genes (*AtATG8a*, *AtATG8e*, *AtATG8f*, *AtATG8g*), respectively, in Arabidopsis could significantly increase the number of autophagosomes and the transcripts of other *AtATG* genes [35]. Under full N conditions, although N remobilization efficiency significantly increased relative to the control line by ^15^N isotope tracer test, the *ATG8* overexpressors did not affect yield and biomass [35]. We two did similar but different studies during the same period. Although these similar conclusions were got in our research, there were also clear differences between us. It seemed that our *OsATG8b* gene was powerful in the development of plant. The overexpressing *OsATG8b* transgenic Arabidopsis not only unaffected the expression of other *AtATG*s (S2 Fig), but also significantly increased seed yield and thousand grains weight under normal growth conditions. Another point worth mentioned was the nitrogen absent (ND) condition of in our experiment was 0 mM N, it was a very very harsh stress condition. Our transgenic plants were still doing well and made a better state of growth, even restored the growth inhibition caused by N stress (Figs 2 and 3). Although rice and Arabidopsis are two type plants, there may be differences in gene function. In addition, the conclusions of we two groups could support or complement each other.

Taken together, our results suggest that the rice autophagy gene *OsATG8b* that can enhance NUE and improve crop yield, which has a positive role in the maintenance of plant fitness and adaptation to nutrient limitations. These results have also linked autophagy to plant nitrogen distribution/homeostasis, as well as increased yield.

## Materials and methods

### Plant materials and growth conditions

Rice (Japonica cv Shennong9816) seeds were sterilized and germinated at 28°C for 2 d, then grown hydroponically in a growth chamber (28°C/25°C and 10 h light/14 h dark). Arabidopsis seeds (Columbia-0) were surface-sterilized and stratified at 4°C for 2 days in the dark, then grow on ½ Murashige and Skoog (½ MS) medium (with 0.75% agar and 1% sucrose (w:v), pH = 5.7) in a growth chamber (22 ± 1°C and 16 h light/8 h dark). 8-day-old Arabidopsis seedlings were transferred to soil with sufficient nutrient and cultivated under the same conditions.

### Total RNA extraction and gene expression analysis

To analysis *OsATG8b* expression responsive to N limitation in rice, rice seedlings cultured with half Hoagland’s solution (NS) as previous described [30] for 14 days, then transferred to the same solution as control, low N (NL) solution (with 0.6 mM KNO_3_ and 0.1 mM (NH_4_)_2_SO_4_, pH = 5.7) and the N-free (ND) solution (pH = 5.7), the lack of K^+^ was replaced with KCl. After 1 day and 3 days N limitation treatments, the leaves and roots were harvested separately for *OsATG8b* gene expression analysis. 10-day-old seedlings of transgenic lines and WT grown on NS were sampled for expression analysis of the endogenous *AtATG*s by real-time RT-PCR. Total RNA extraction was conducted using the Eastep Super Total RNA Extraction Kit (Promega) and first strand cDNA was synthesized with the PrimeScript RT Master Mix (TaKaRa). Real-time RT-PCR was performed as described previously [51] by using SYBR Premix Ex TaqII (TaKaRa) on an Applied Biosystems 7500 Real Time PCR System. The following standard thermal profile was used for all PCRs: 95°C for 30 s, 40 cycles of 95°C for 5 s and 60°C for 34 s. All reactions were done at least in triplicates. *TIP41* was used as an internal control. The primers used for RT-PCR analysis were listed in S1 Table.

### Binary vector construction and Arabidopsis genetic transformation

The complete coding region of *OsATG8b* was amplified by PCR using a pair of primers with full-length cDNA of rice as the template. The PCR product was then ligated into binary vector pCAMBIA1301, resulting in the *35S-OsATG8b* fusion gene. For the *Pro*_*OsATG8b*_*-GUS* construct, a 1988-bp promoter fragment of the *OsATG8b* was amplified from rice genomic DNA by PCR, and then ligated into binary vector pCAMBIA1301. All the construction was confirmed by DNA sequencing. The plant expression vector was transformed into Arabidopsis by *Agrobacterium*-mediated floral dipping method [52]. All the primers used were listed in S1 Table. T_0_ transgenic Arabidopsis seeds were screened on ½ MS medium containing 30 mg·L^-1^ hygromycin and seedlings with green true leaves were identified as transformants, 3 independents homozygous T_3_ lines were analyzed in this study.

### Protein isolation and immunoblot analysis

Total proteins extraction was conducted using the Minute^™^ Total Protein Extraction Kit (Invent Biotechnologies, Inc). Total membrane protein extraction was conducted using the Minute™ Membrane Protein Isolation Kit for plants (Invent Biotechnologies, Inc). 14-day-old seedlings of transgenic lines and WT grown on NS were sampled to exam the protein level of OsATG8b, the full length OsATG8b was used to raise a polyclonal antibody (GenScript, Nanjing China). Protein concentration was determined with the BCA Protein Assay Kit (TaKaRa). For autophagic flux analysis, 9-day-old seedlings of transgenic lines and WT were transferred to NS or ND medium with 0.5 μM ConA or solvent control DMSO for 24h. All SDS-PAGE gels were prepared with 6 M urea, antibodies against Arabidopsis anti-ATG8a and anti-NBR1 (1:1000), anti-α-tubulin (1:1000) (Agirsera, Sweden) and anti-OsATG8b (1:1000) were used as primary antibodies; goat anti-rabbit (IgG) (Sigma-Aldrich) was used as the secondary antibody. Immunoblotting were done as described [28,30]. The protein band intensities of immunoblots was analyzed by Image J software. Each experiment was repeated for three times, and one representative result was shown.

### Phenotypic analysis of *OsATG8b* transgenic Arabidopsis under optimum condition and N starvation

For the basal phenotypic analysis of transgenic lines and the WT under sufficient nutrient condition, 8-day-old (days after germination) seedlings were transferred to soil. The maximum rosette radius and the plant height were recorded every 3 days, the bolting and flowering times were recorded every day from Stage5.1 and Stage6.0 [53], respectively. The leaf number at bolting was counted when the inflorescence emergence at the Stage5.1, and the total leaf number was counted after the rosettes growth completed at about Stage6.0. Total number of siliques was counted when the plants growth to Stage6.9, the total seeds produced by a single-plant were harvested at the growth Stage9.7, and the yield per plant was expressed as the weight of total seeds per plant, and the thousand-grain weight were weighed. For N starvation treatments, 7-day-old seedlings of transgenic lines and WT were transferred to NS or ND for 9 days. The phenotypic changes were recorded by scanner (EPSON Perfection v33), the fresh weight of leaves was measured.

### GUS histochemical staining

Seeds of *Pro*_*OsATG8b*_*-GUS* transgenic Arabidopsis were screened on ½ MS medium containing 30 mg·L^-1^ hygromycin for temporal and spatial expression patterns analysis, then GUS histochemical staining was conducted as described [54].

### MDC staining for detecting autophagosome activity

7-day-old seedlings of transgenic lines and WT were transferred to ND liquid medium containing 1 μmol·L^-1^ ConA with or without 5 mM 3-MA for 12h, then the leaves were excised and immediately vacuum infiltrated with 50 mM MDC (Sigma-Aldrich) for 10 min, MDC staining was conducted as described [48], and confocal images were acquired using an inverted Zeiss LSM 780 laser scanning microscope. Quantification of the MDC-stained autophagosomes in leaves was presented as the fluorescence ratio = positive signal/area. The number of positive signals was calculated from areas of 400 μm^2^, and more than 10 independent areas were used for the quantification.

### Measurement of chlorophyll, soluble proteins, and nitrate reductase (NR) enzyme activity

Chlorophyll content was measured with Arnon method as describe [55]. Soluble protein content was determined with the Bradford method [56]. The NR enzyme activity was assayed as described previously [57].

### Biomass, total N, C concentrations and indicator used to monitor NUE calculations

Plants were sampled by separation into rosettes (including cauline leaves), stems, and seeds at harvest, then all samples were kept in a dry oven at 80°C for 7 days, and dry mass were weighted as DW_Dr_ (dry weight of rosettes and stems) and DW_Seeds_ (dry weight of total seeds). Then the dry mass of all these tissue organs were ground to powder, total N and C concentrations (N% and C%) were measured using an Elemental Analyzer (Elementa, Vario MAX C, Germany), which presented as N% (C%) = mg N (C)/100 mg DW. Biomass was calculated as DW_Dr_+DW_Seeds_. Indicator used to monitor NUE were calculated as described [20]. The harvest index (HI) was calculated as DW_Seeds_/(DW_Seeds_+DW_Dr_) ratio and was used as an important indicator for yield. The N harvest index (NHI) was used to evaluate grain N filling and was calculated as (N%_Seeds_×DW_Seeds_)/(N%_Seeds_×DW_Seeds_+N%_Dr_×DW_Dr_). The ratio of NHI/HI was estimated as the percentage of WT to compare the NUE performance among genotypes.

### ^15^N-Labeling and NRE calculation

The ^15^N-Labeling experiment was conducted as Guiboileau et al (2012) [20] described on WT and *35S-OsATG8b* transgenic lines at Stage5.1 (long time before flowering), the unlabeled watering solutions was replaced with one containing the same nutrient composition except that plants were supplied with ^15^NH_4_ and ^15^NO_3_ (10.6 atom% excess) for 3 d. Afterwards, the pots were rinsed thoroughly with distilled water to remove the remaining ^15^N, and unlabeled watering solutions were then applied until seed harvest. When the plants were grown to maturity, the plants were separated into remains [rosettes (including cauline leaves) and stems] and seeds. All samples were kept in a dry oven at 80°C for 7 days, then dry mass was weighted and ground to fine powder to assess N remobilization using an elemental analyzer/isotope ratio mass spectrometry system (Thermo Scientific, Flash2000/Delta V Advantage), and unlabeled plants were also collected to determine the natural ^15^N abundance. The ^15^N abundance of each sample was expressed as a percent of total N and calculated as atom% sample (A%sample), A%sample = 100×(^15^N)/(^15^N+^14^N). The natural abundance of ^15^N (Acontrol%) obtained from unlabeled samples were considered using the average value 0.365% to calculate ^15^N enrichment of labeled samples (E%), E% = A%sample-A%control. NRE in this experiment were estimated as described by Guiboileau et al (2012) [20], using the ratio of ^15^NHI/HI, and they were estimated as the percentage of WT to compare the NRE performance among genotypes. ^15^NHI was calculated as (E%_Seeds_×N%_Seeds_×DW _Seeds_)/[(E%_Seeds_×N%_Seeds_×DW_Seeds_)+(E%_Dr_×N%_Dr_×DW_Dr_)].

### Statistical analysis

The experiments were repeated three times, and data from one representative experiment were shown here. The statistically significant differences of all data were calculated based on Student's t-test at significance levels of *P < 0.05 and **P < 0.01.

## Supporting information

S1 Fig GUS histochemical staining of *Pro*_*OsATG8b*_*-GUS* transgenic Arabidopsis at different developmental stages. (A) Seeds in germination; (B) 1-week-old seedlings; (C) 2-week-old seedlings; (D) 4-week-old of mature seedlings; (E) Early inflorescence; (F) Mature inflorescences and siliques. The left is transgenic Arabidopsis before staining, the right is that after staining. (A) and (B) Bars = 1 mm; (C) and (F) Bars = 5 mm.
(TIF)

S2 Fig Real-time RT-PCR analysis of the transcript levels of *AtATG*s in 14-day-old seedlings of WT and *35S-OsATG8b* transgenic Arabidopsis under N sufficient condition. (TIF)

S3 Fig Molecular analysis of transgenic lines. PCR identification of genomic. DNA of transgenic lines. M, Molecular marker DL2000; 1–9, Independent transgenic lines; +, Positive vector containing 35S-OsATG8b plasmid; -, Negative control.
(TIF)

S1 Table The information of primers used in this study. (DOCX)
