# Supplementary figures and images for: Overexpression of rice gene OsATG8b confers tolerance to nitrogen starvation and increases yield and nitrogen use efficiency (NUE) in Arabidopsis
Source: PLoS One. 2019 Sep 25;14(9):e0223011. doi: 10.1371/journal.pone.0223011 (PMC6760796; doi:10.1371/journal.pone.0223011)

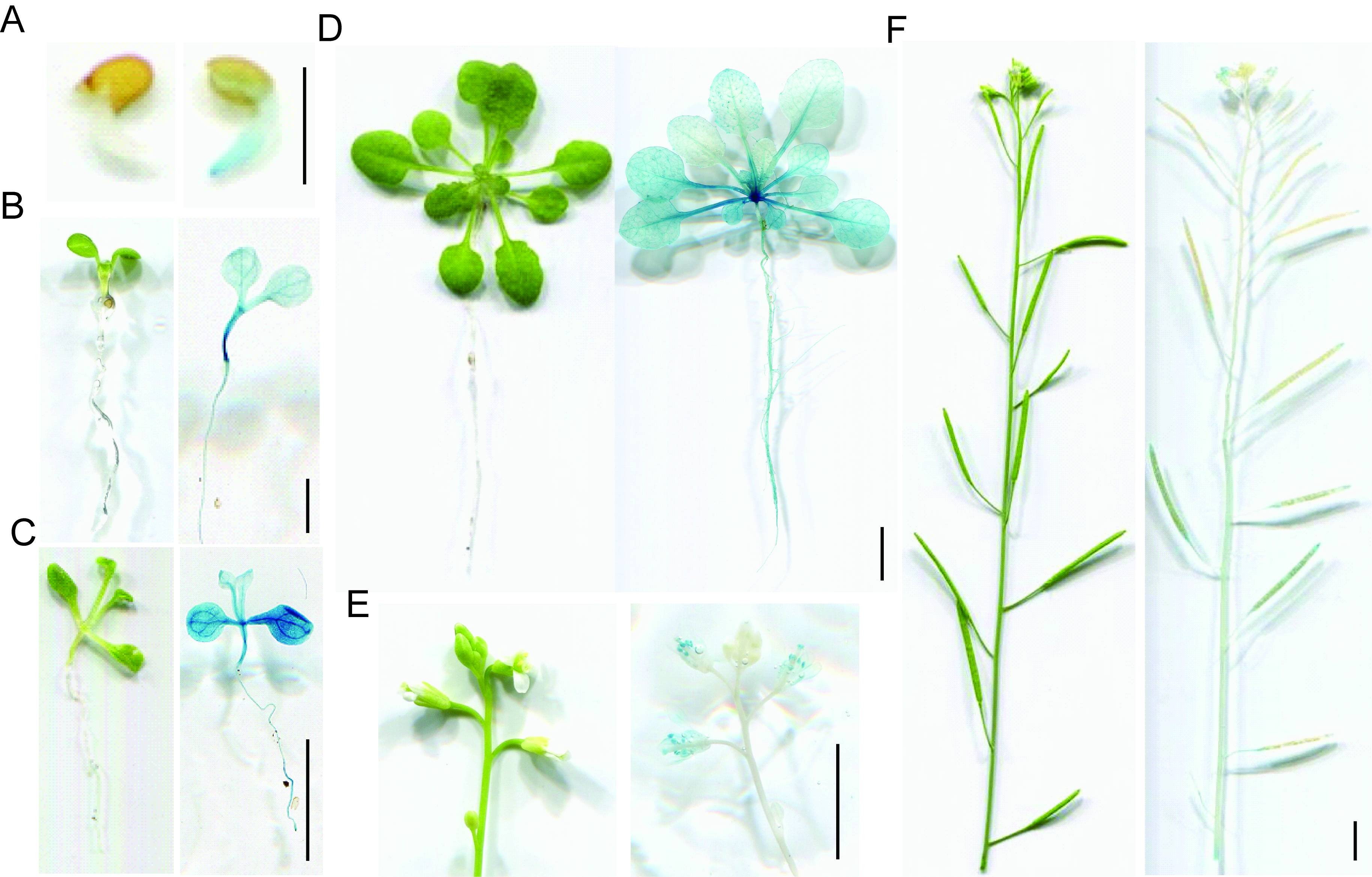

Supplement: S1 Fig — (A) Seeds in germination; (B) 1-week-old seedlings; (C) 2-week-old seedlings; (D) 4-week-old of mature seedlings; (E) Early inflorescence; (F) Mature inflorescences and siliques. The left is transgenic Arabidopsis before staining, the right is that after staining. (A) and (B) Bars = 1 mm; (C) and (F) Bars = 5 mm. (TIF) [file pone.0223011.s001.tif]

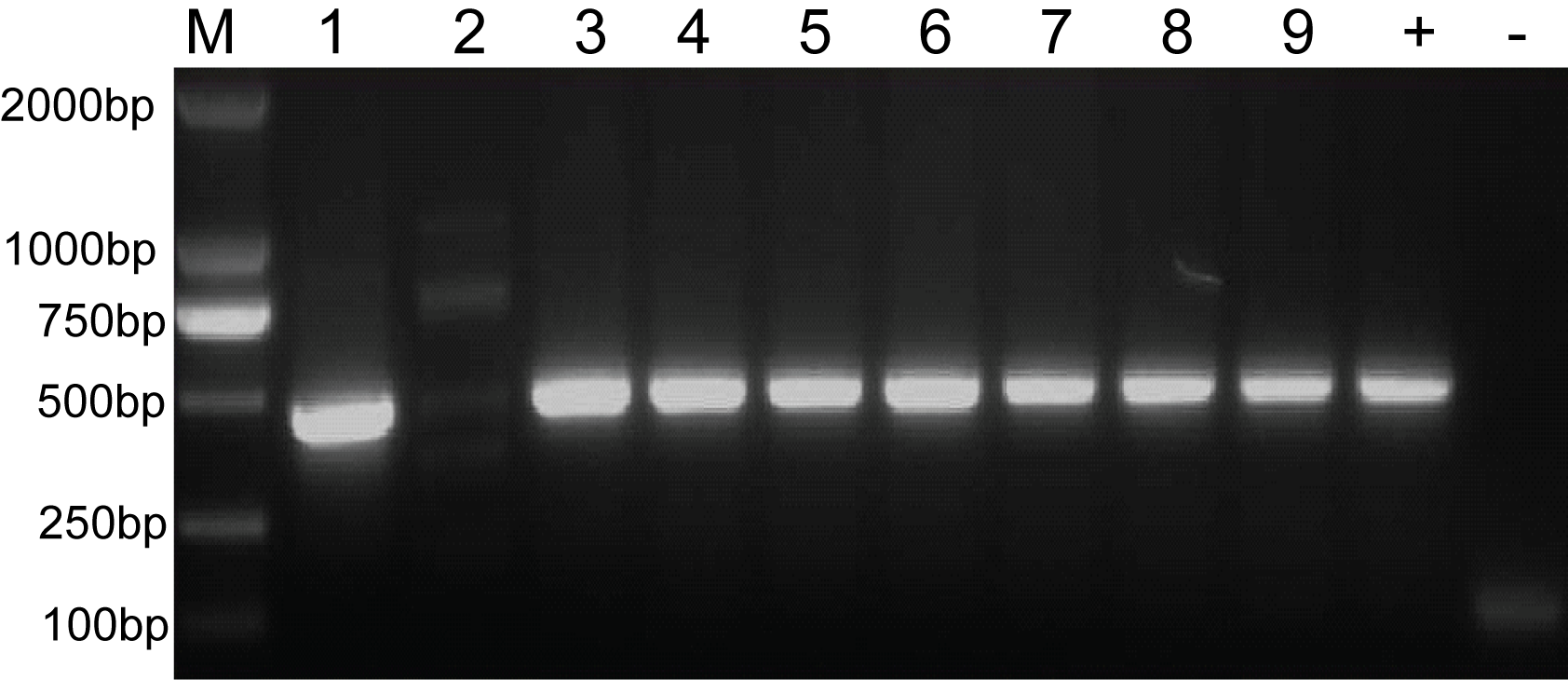

Supplement: S3 Fig — PCR identification of genomic. DNA of transgenic lines. M, Molecular marker DL2000; 1–9, Independent transgenic lines; +, Positive vector containing 35S-OsATG8b plasmid; -, Negative control. (TIF) [file pone.0223011.s003.tif]
