# Supplementary material for: Overexpression of rice gene OsATG8b confers tolerance to nitrogen starvation and increases yield and nitrogen use efficiency (NUE) in Arabidopsis
Source: PLoS One. 2019 Sep 25;14(9):e0223011. doi: 10.1371/journal.pone.0223011 (PMC6760796; doi:10.1371/journal.pone.0223011)
Supplement: S1 Table — (DOCX) [file pone.0223011.s004.docx]

| Primer name | Sequence (5´→ 3´) | Functions |
| --- | --- | --- |
| cOsATG8b-F | CCATTCAAGTGGATGGCCAAGAGCTCGTTCAAGC | Gene cloning |
| cOsATG8b-R | GGTGACCTAGAGCAGCCCAAAGGTGTTCTCG | Gene cloning |
| cpOsATG8b-F | AAGCTTAAAATTAAATAAGACGAACAGTCAAACG | Gene cloning |
| cpOsATG8b-R | CCATGGCGCTCCTTCCTGCACACAAT | Gene cloning |
| rtOsATG8b-F | GCTGATCTTACCGTTGGGCA | Real-time RT-PCR |
| rtOsATG8b-R | ATCAGAGCAGCTGTTGGTGG | Real-time RT-PCR |
| rtActin1-F | ACCATTGGTGCTGAGCGTTT | Real-time RT-PCR |
| rtActin1-R | CGCAGCTTCCATTCCTATGAA | Real-time RT-PCR |
| rtTIP41-F | GTATGAAGATGAACTGGCTGACAAT | Real-time RT-PCR |
| rtTIP41-R | ATCAACTCTCAGCCAAAATCGCAAG | Real-time RT-PCR |

**S1 Table.** The information of primers used in this study.
